# Supplementary material for: Rapid DNA methylation-based classification of pediatric brain tumors from ultrasonic aspirate specimens
Source: J Neurooncol. 2024 May 21;169(1):73–83. doi: 10.1007/s11060-024-04702-6 (PMC11269392; doi:10.1007/s11060-024-04702-6)
Supplement: Supplementary file 1 — Supplementary file1 Comparison of copy number variation profiles obtained from ultrasonic aspirator tissue samples and nanopore sequencing versus FFPE tumor tissue subjected to EPIC microarray (850K). (PDF 4486 KB) [file 11060_2024_4702_MOESM1_ESM.pdf]

Supplemental Figure 1: Comparison of copy number variation profiles obtained from **(A)** ultrasonic aspirator tissue samples and nanopore sequencing and **(B)** FFPE tumor tissue subjected to EPIC microarray (850K).

(A)

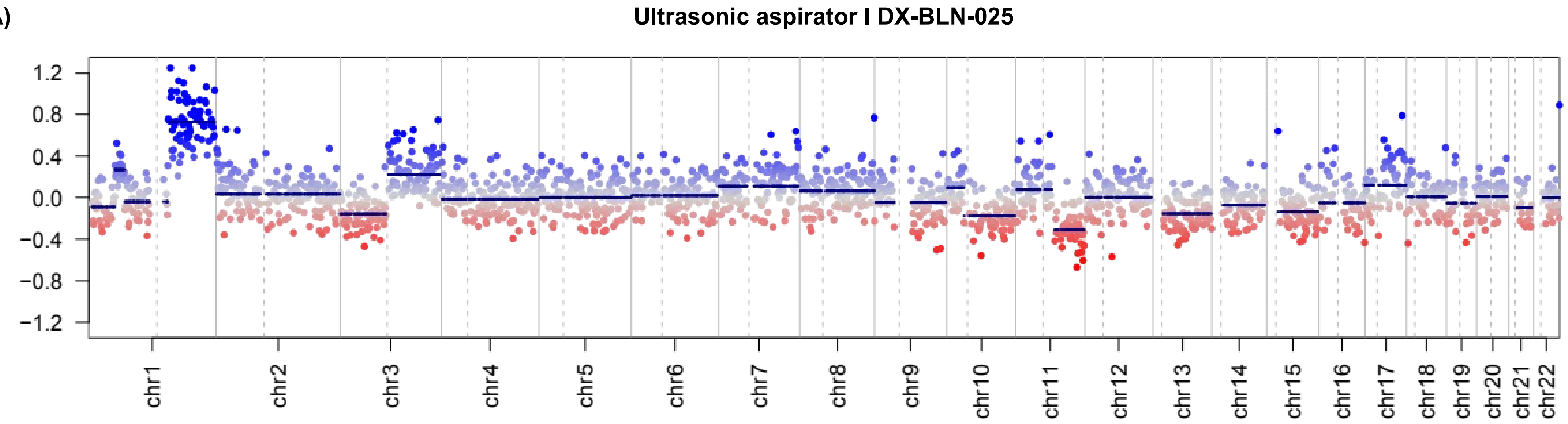

(B)

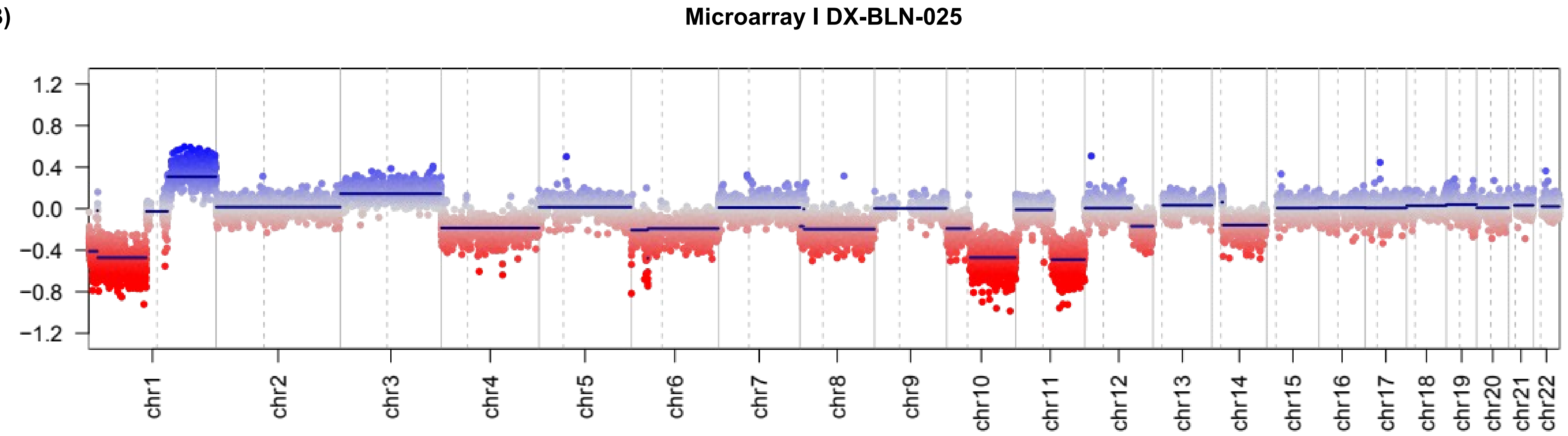

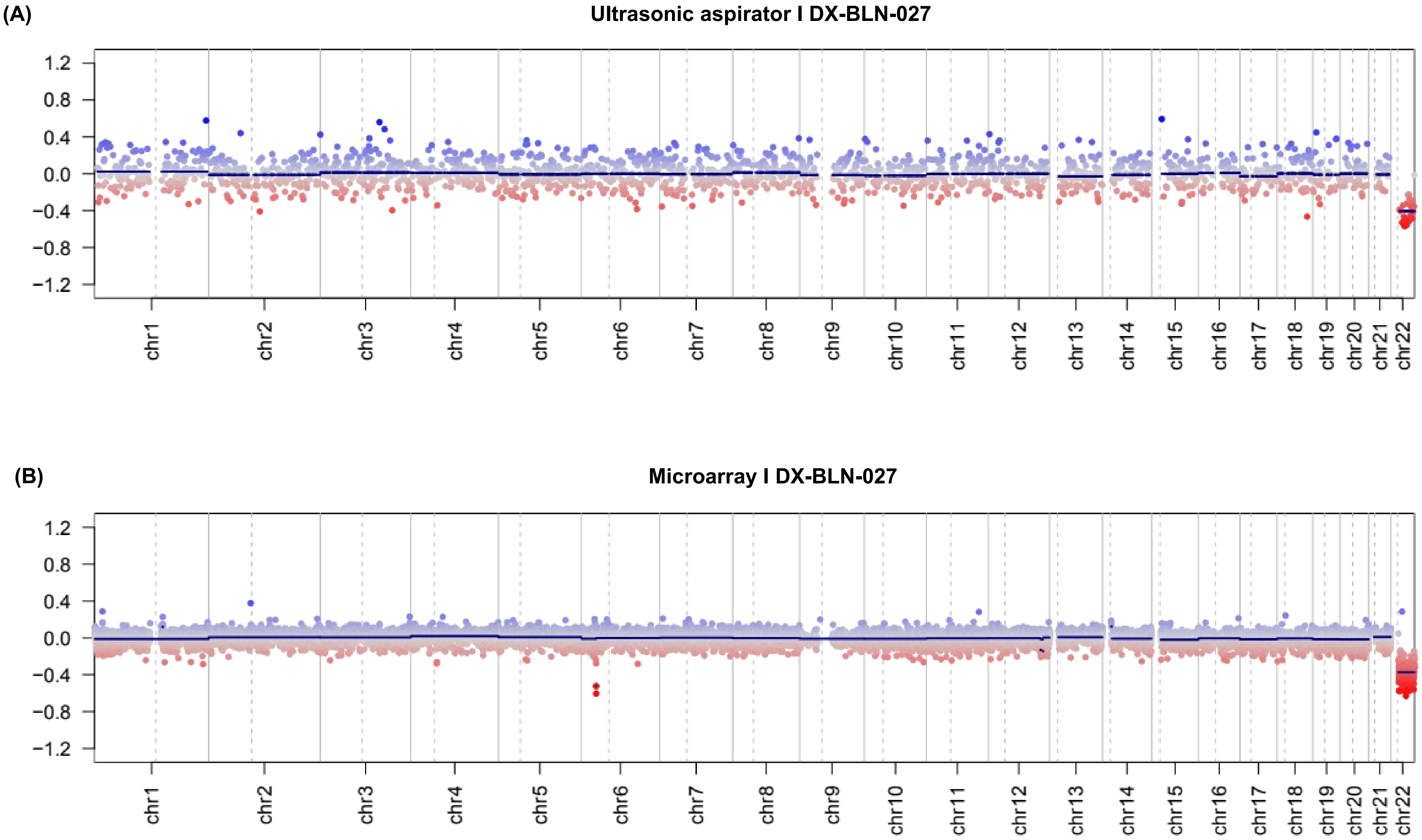
(A)
Ultrasonic aspirator I DX-BLN-027
1.2
0.8
0.4
0.0
−0.4
−0.8
−1.2
chr1 chr2 chr3 chr4 chr5 chr6 chr7 chr8 chr9 chr10 chr11 chr12 chr13 chr14 chr15 chr16 chr17 chr18 chr19 chr20 chr21 chr22
(B)
Microarray I DX-BLN-027
1.2
0.8
0.4
0.0
−0.4
−0.8
−1.2
chr1 chr2 chr3 chr4 chr5 chr6 chr7 chr8 chr9 chr10 chr11 chr12 chr13 chr14 chr15 chr16 chr17 chr18 chr19 chr20 chr21 chr22

(A)

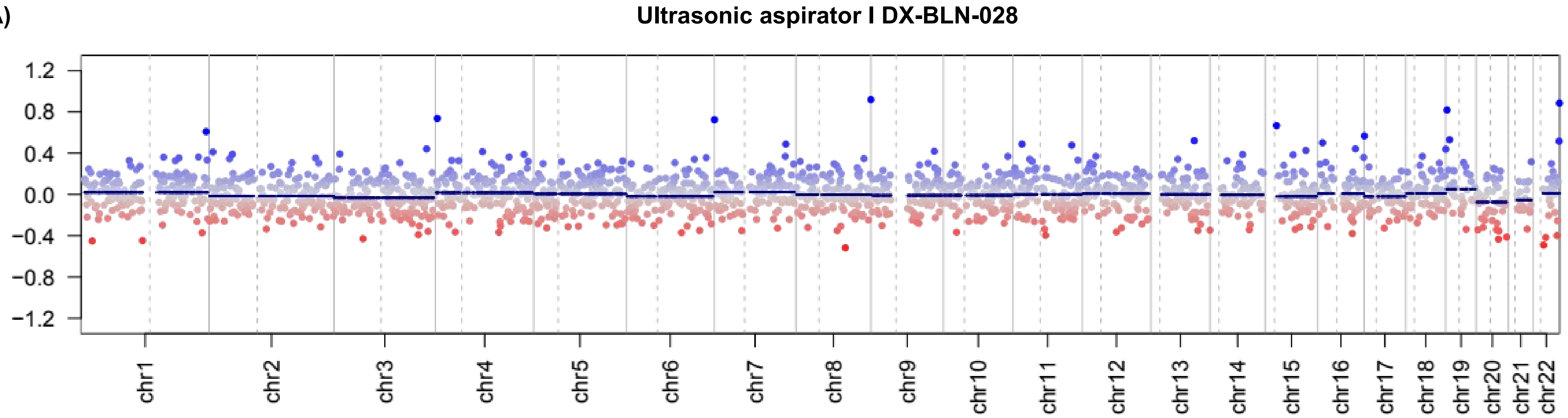
Ultrasonic aspirator I DX-BLN-028
1.2
0.8
0.4
0.0
−0.4
−0.8
−1.2
chr1
chr2
chr3
chr4
chr5
chr6
chr7
chr8
chr9
chr10
chr11
chr12
chr13
chr14
chr15
chr16
chr17
chr18
chr19
chr20
chr21
chr22

(B)

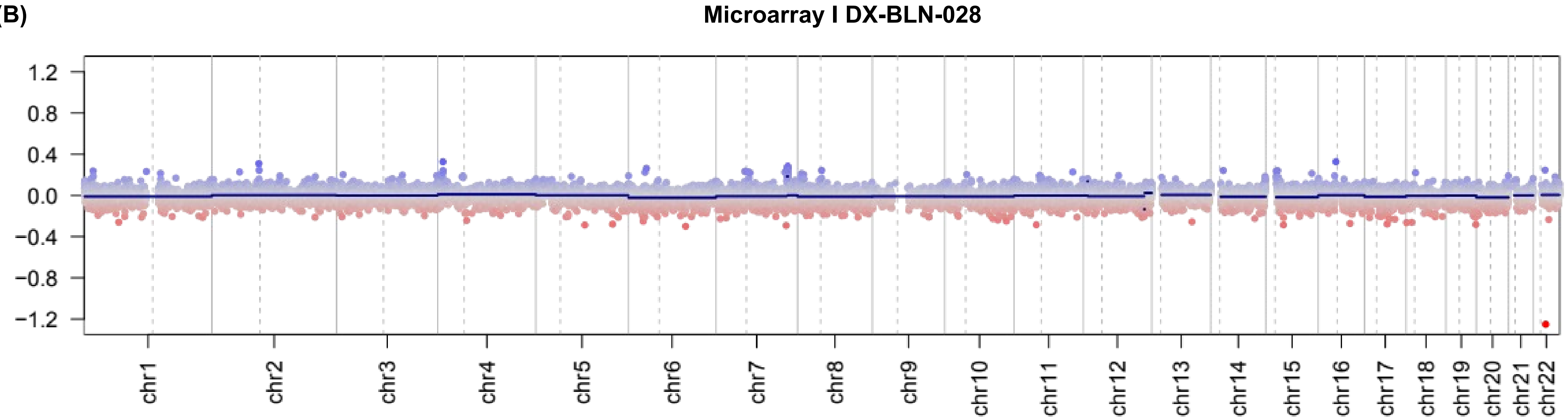
Microarray I DX-BLN-028
1.2
0.8
0.4
0.0
−0.4
−0.8
−1.2
chr1
chr2
chr3
chr4
chr5
chr6
chr7
chr8
chr9
chr10
chr11
chr12
chr13
chr14
chr15
chr16
chr17
chr18
chr19
chr20
chr21
chr22

(A)

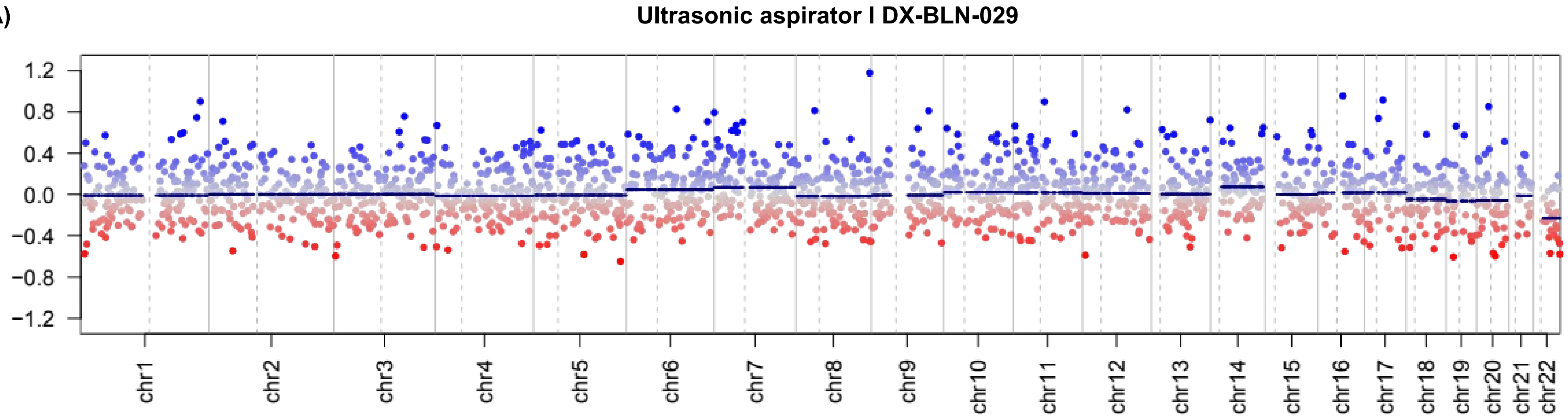
Ultrasonic aspirator I DX-BLN-029
1.2
0.8
0.4
0.0
−0.4
−0.8
−1.2
chr1
chr2
chr3
chr4
chr5
chr6
chr7
chr8
chr9
chr10
chr11
chr12
chr13
chr14
chr15
chr16
chr17
chr18
chr19
chr20
chr21
chr22

(B)

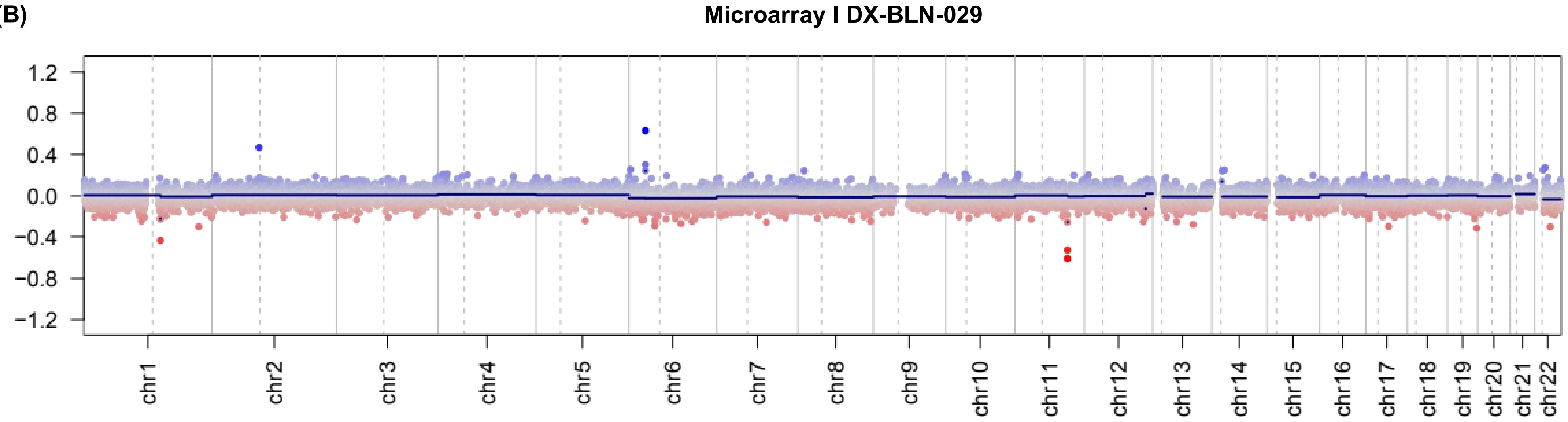
Microarray I DX-BLN-029
1.2
0.8
0.4
0.0
−0.4
−0.8
−1.2
chr1
chr2
chr3
chr4
chr5
chr6
chr7
chr8
chr9
chr10
chr11
chr12
chr13
chr14
chr15
chr16
chr17
chr18
chr19
chr20
chr21
chr22

(A)

Ultrasonic aspirator I DX-BLN-037

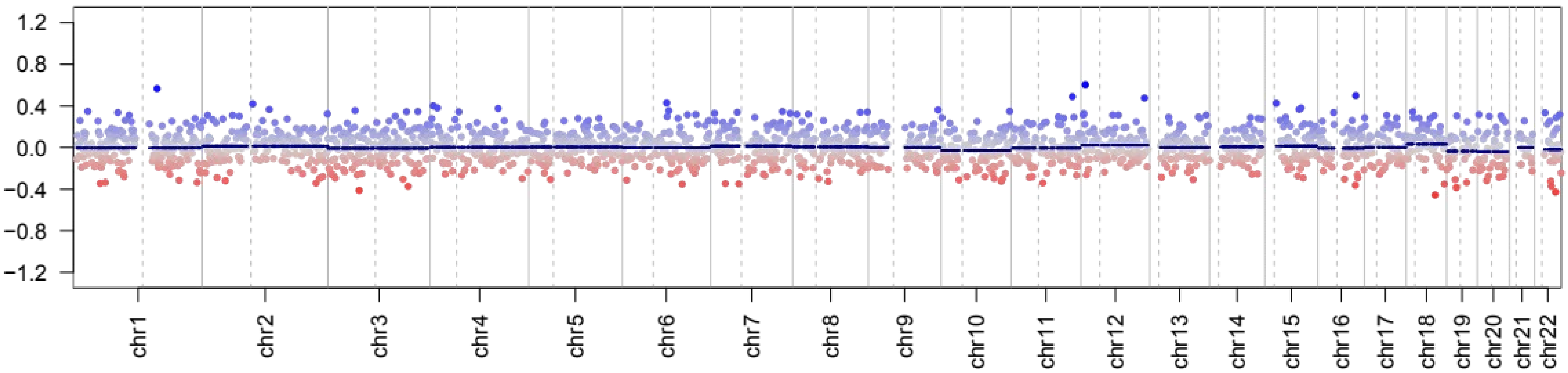

(B)

Microarray I DX-BLN-037

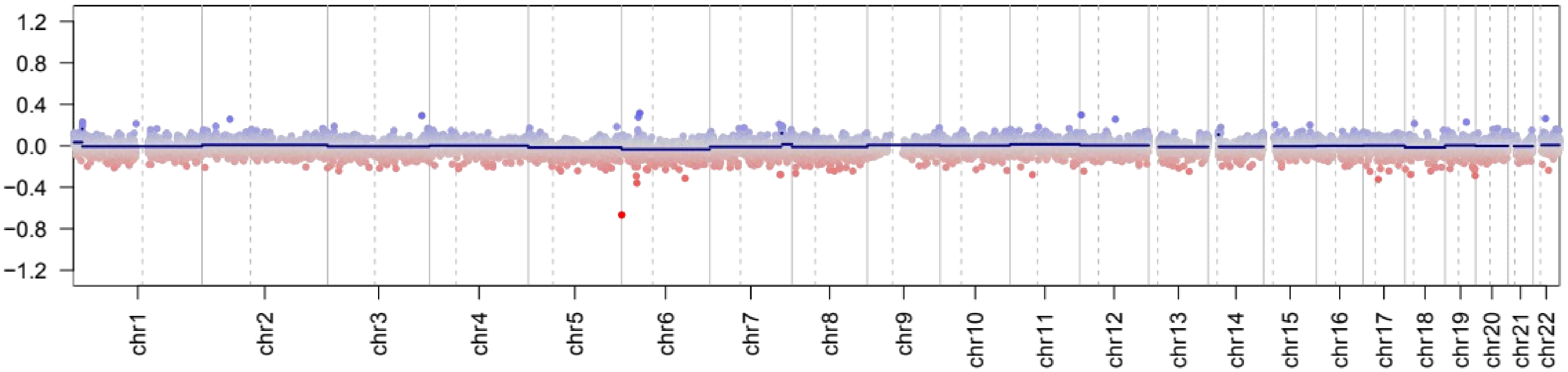

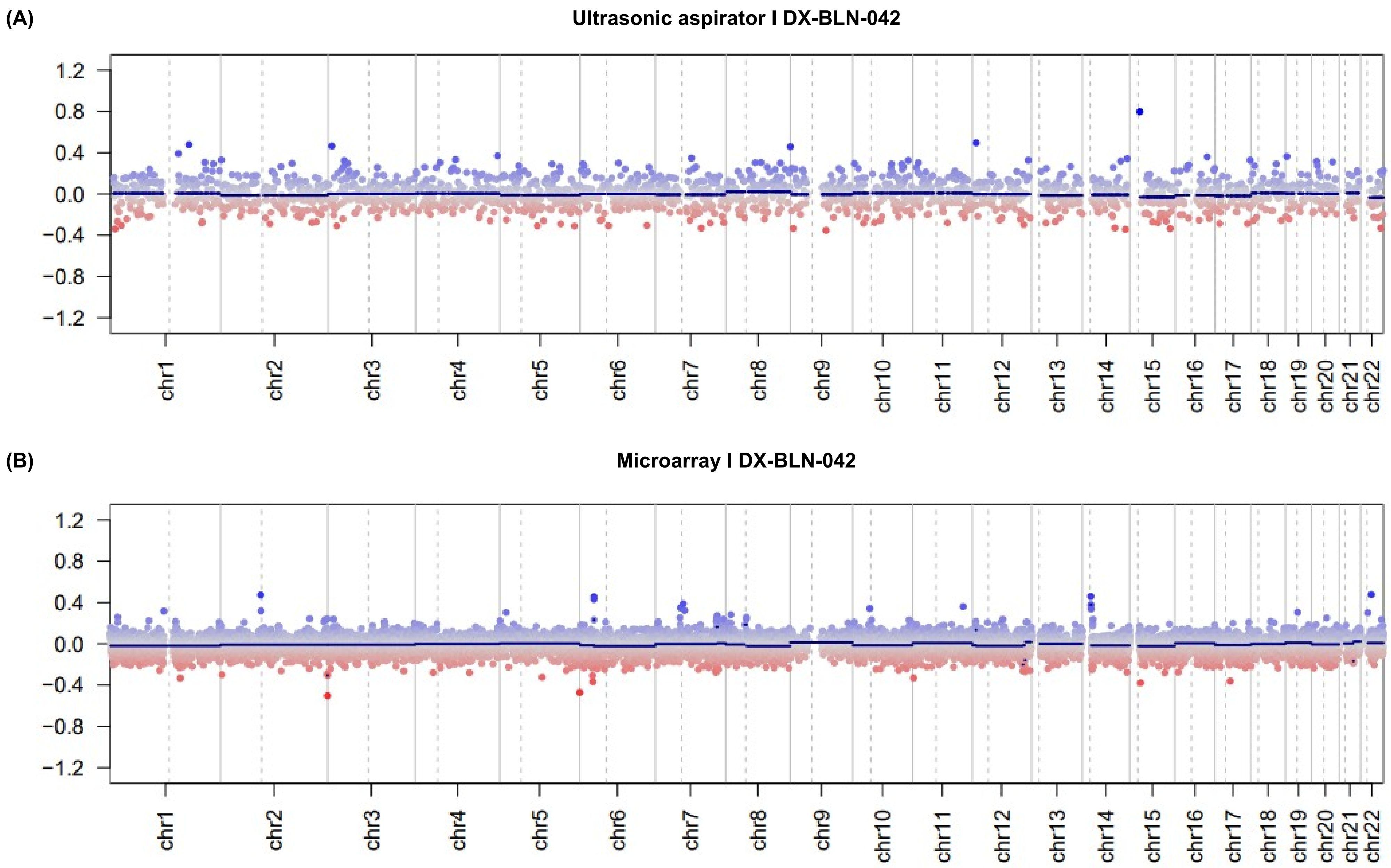
(A)
Ultrasonic aspirator I DX-BLN-042
1.2
0.8
0.4
0.0
−0.4
−0.8
−1.2
chr1 chr2 chr3 chr4 chr5 chr6 chr7 chr8 chr9 chr10 chr11 chr12 chr13 chr14 chr15 chr16 chr17 chr18 chr19 chr20 chr21 chr22
(B)
Microarray I DX-BLN-042
1.2
0.8
0.4
0.0
−0.4
−0.8
−1.2
chr1 chr2 chr3 chr4 chr5 chr6 chr7 chr8 chr9 chr10 chr11 chr12 chr13 chr14 chr15 chr16 chr17 chr18 chr19 chr20 chr21 chr22

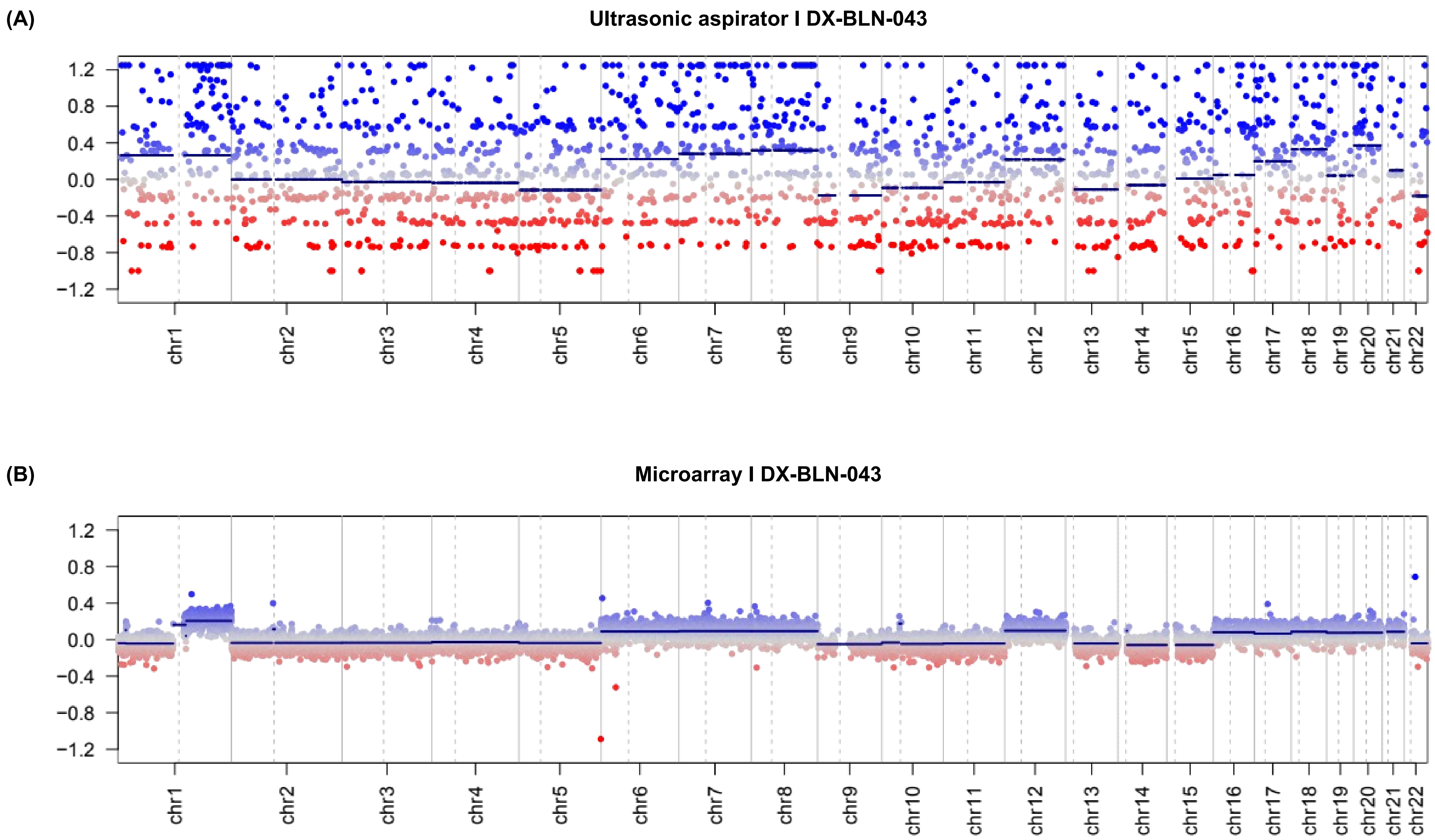
(A)
Ultrasonic aspirator I DX-BLN-043
1.2
0.8
0.4
0.0
−0.4
−0.8
−1.2
chr1 chr2 chr3 chr4 chr5 chr6 chr7 chr8 chr9 chr10 chr11 chr12 chr13 chr14 chr15 chr16 chr17 chr18 chr19 chr20 chr21 chr22
(B)
Microarray I DX-BLN-043
1.2
0.8
0.4
0.0
−0.4
−0.8
−1.2
chr1 chr2 chr3 chr4 chr5 chr6 chr7 chr8 chr9 chr10 chr11 chr12 chr13 chr14 chr15 chr16 chr17 chr18 chr19 chr20 chr21 chr22

(A)

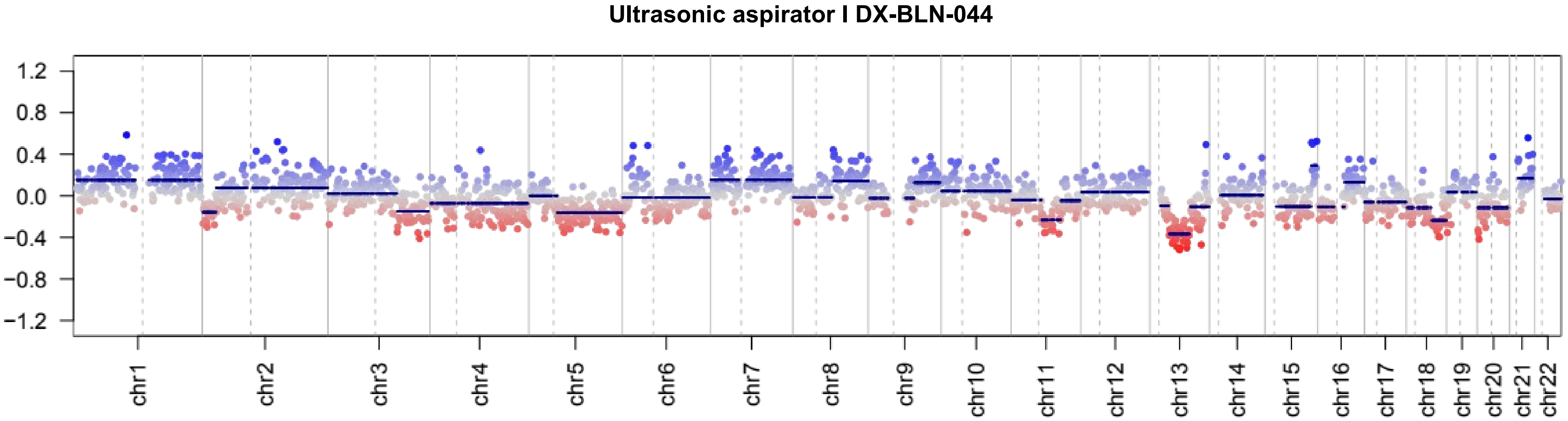
Ultrasonic aspirator I DX-BLN-044
1.2
0.8
0.4
0.0
−0.4
−0.8
−1.2
chr1 chr2 chr3 chr4 chr5 chr6 chr7 chr8 chr9 chr10 chr11 chr12 chr13 chr14 chr15 chr16 chr17 chr18 chr19 chr20 chr21 chr22

(B)

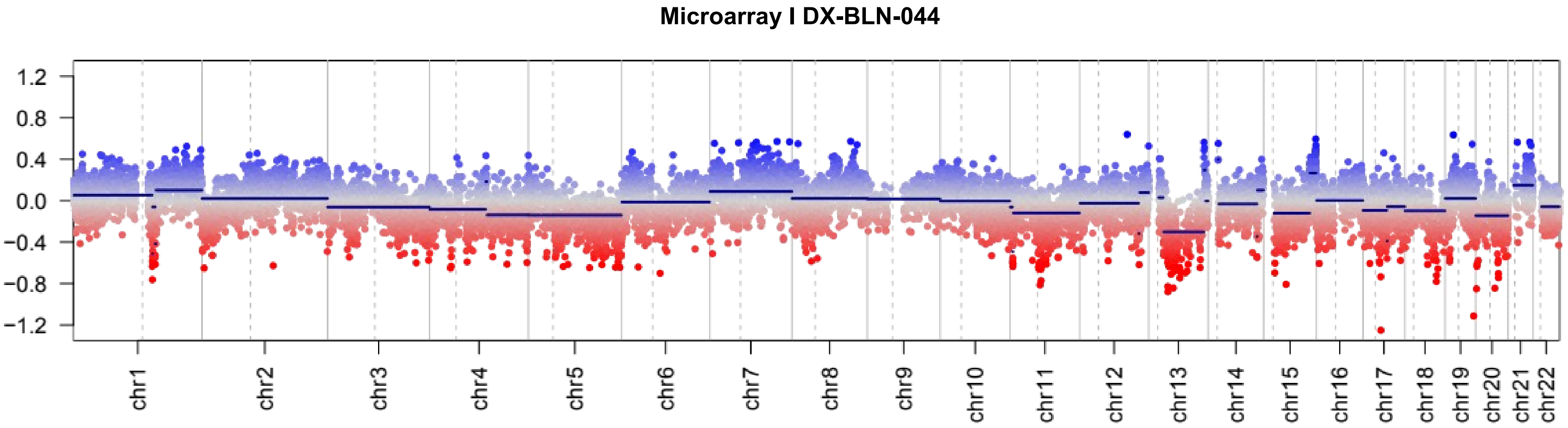
Microarray I DX-BLN-044
1.2
0.8
0.4
0.0
−0.4
−0.8
−1.2
chr1 chr2 chr3 chr4 chr5 chr6 chr7 chr8 chr9 chr10 chr11 chr12 chr13 chr14 chr15 chr16 chr17 chr18 chr19 chr20 chr21 chr22

(A)

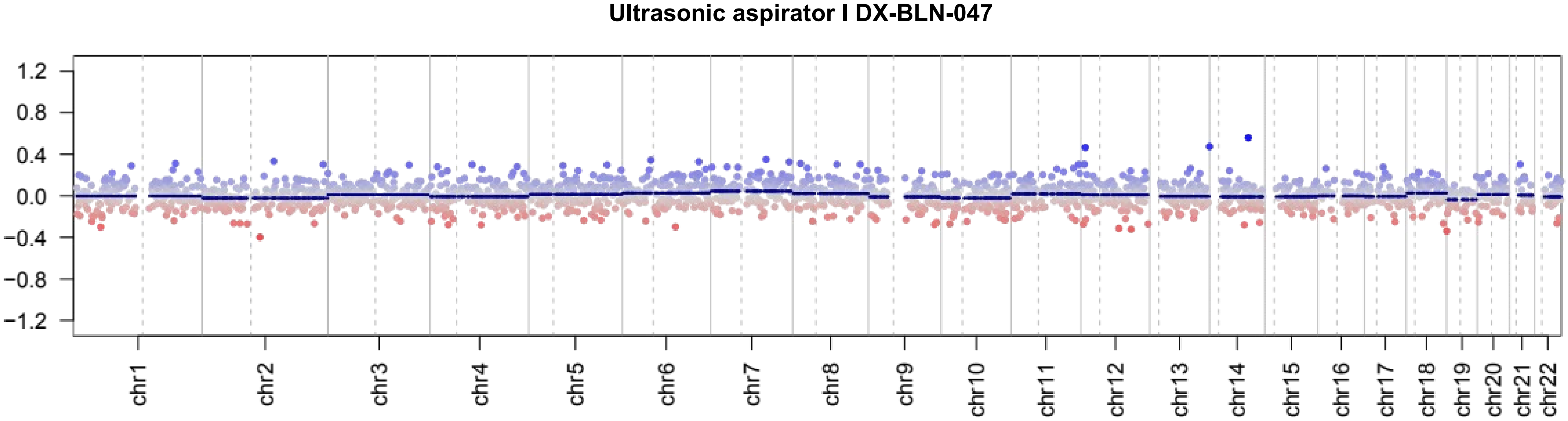
Ultrasonic aspirator I DX-BLN-047
1.2
0.8
0.4
0.0
−0.4
−0.8
−1.2
chr1
chr2
chr3
chr4
chr5
chr6
chr7
chr8
chr9
chr10
chr11
chr12
chr13
chr14
chr15
chr16
chr17
chr18
chr19
chr20
chr21
chr22

(B)

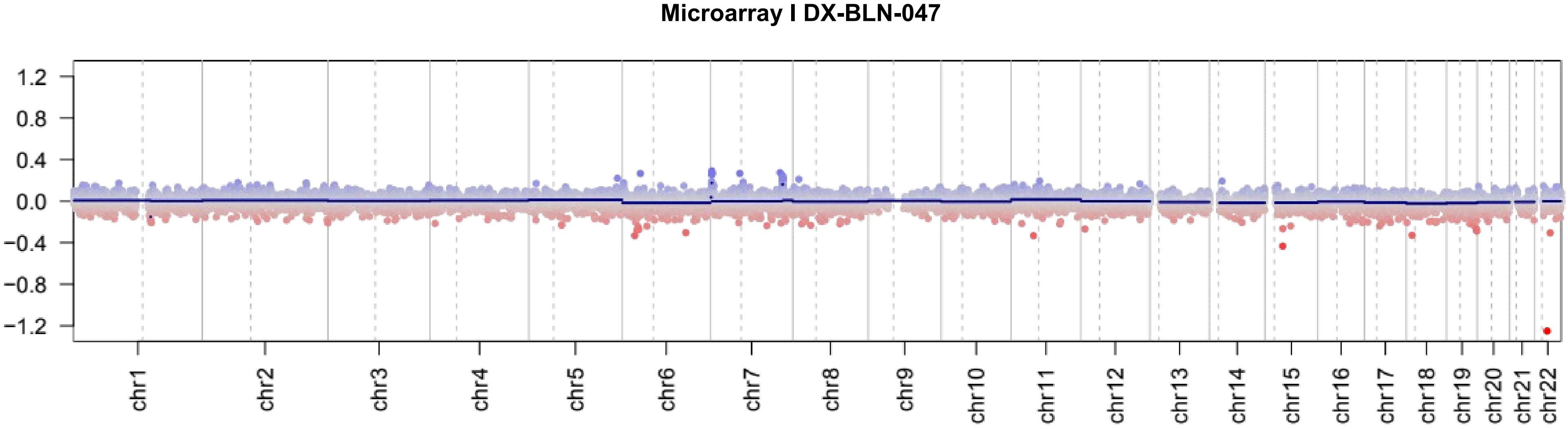
Microarray I DX-BLN-047
1.2
0.8
0.4
0.0
−0.4
−0.8
−1.2
chr1
chr2
chr3
chr4
chr5
chr6
chr7
chr8
chr9
chr10
chr11
chr12
chr13
chr14
chr15
chr16
chr17
chr18
chr19
chr20
chr21
chr22

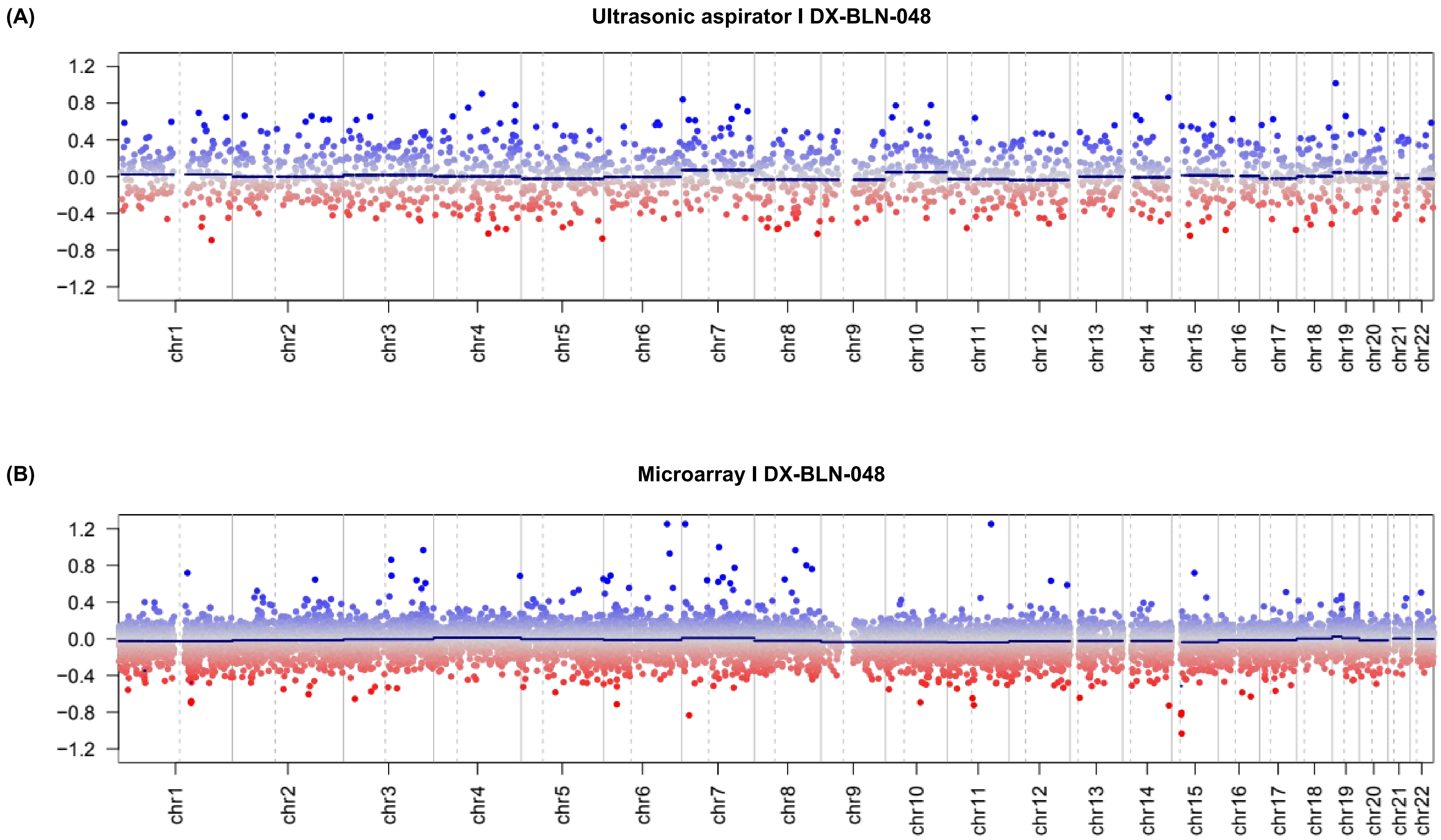
(A)
Ultrasonic aspirator I DX-BLN-048
1.2
0.8
0.4
0.0
−0.4
−0.8
−1.2
chr1
chr2
chr3
chr4
chr5
chr6
chr7
chr8
chr9
chr10
chr11
chr12
chr13
chr14
chr15
chr16
chr17
chr18
chr19
chr20
chr21
chr22
(B)
Microarray I DX-BLN-048
1.2
0.8
0.4
0.0
−0.4
−0.8
−1.2
chr1
chr2
chr3
chr4
chr5
chr6
chr7
chr8
chr9
chr10
chr11
chr12
chr13
chr14
chr15
chr16
chr17
chr18
chr19
chr20
chr21
chr22

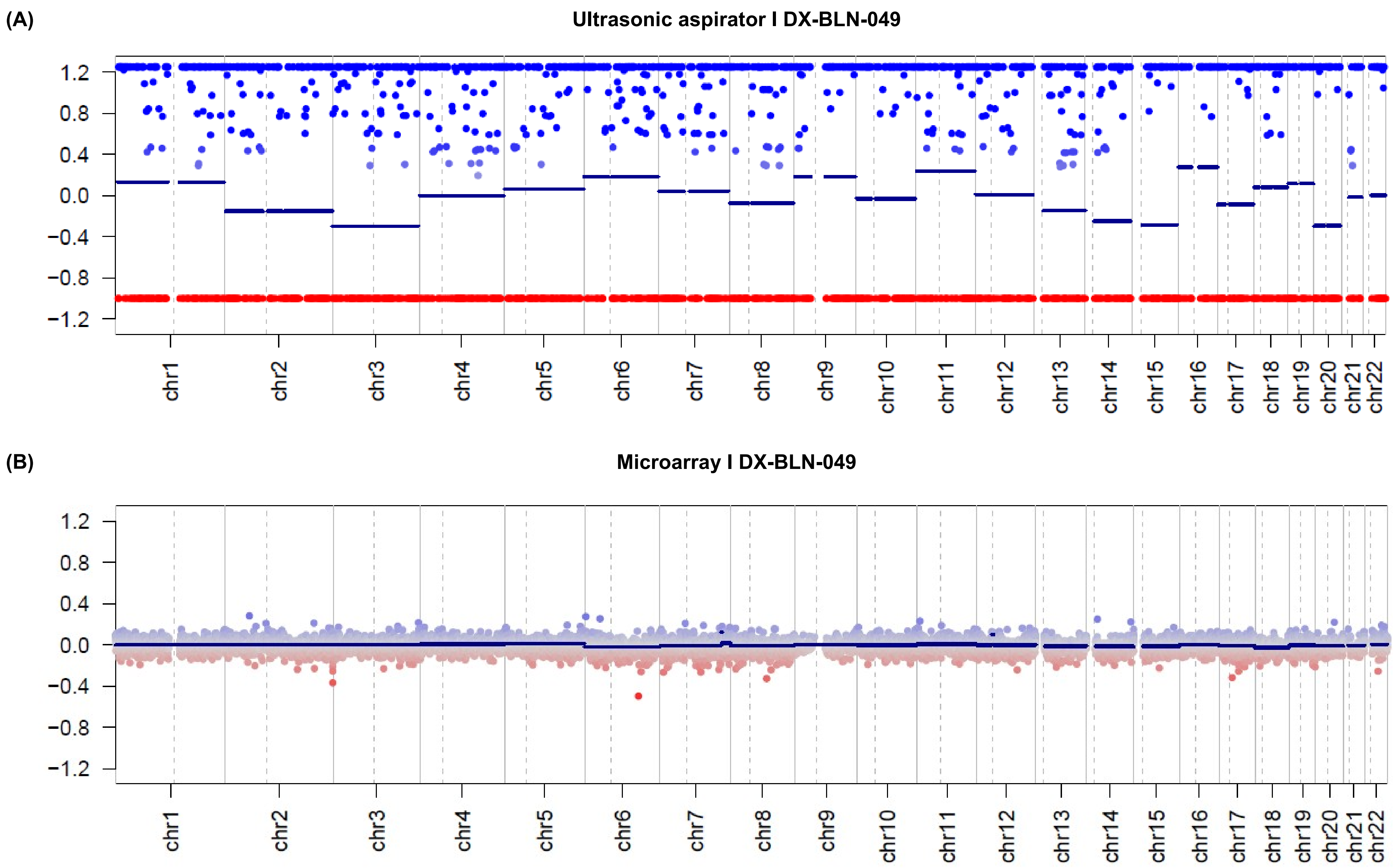
(A)
Ultrasonic aspirator I DX-BLN-049
1.2
0.8
0.4
0.0
−0.4
−0.8
−1.2
chr1 chr2 chr3 chr4 chr5 chr6 chr7 chr8 chr9 chr10 chr11 chr12 chr13 chr14 chr15 chr16 chr17 chr18 chr19 chr20 chr21 chr22
(B)
Microarray I DX-BLN-049
1.2
0.8
0.4
0.0
−0.4
−0.8
−1.2
chr1 chr2 chr3 chr4 chr5 chr6 chr7 chr8 chr9 chr10 chr11 chr12 chr13 chr14 chr15 chr16 chr17 chr18 chr19 chr20 chr21 chr22

(A) **Ultrasonic aspirator I DX-BLN-050**

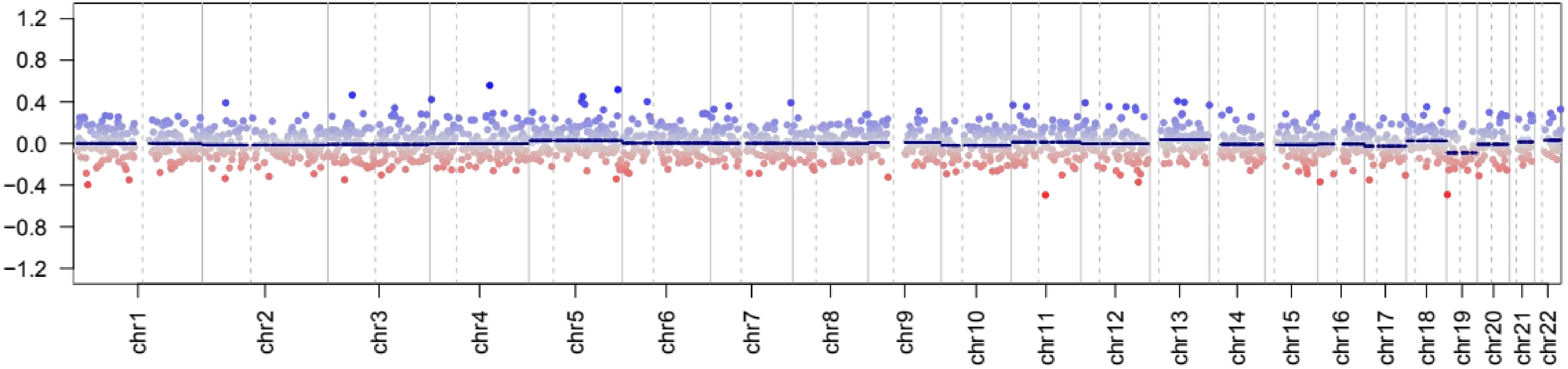

(B) **Microarray I DX-BLN-050**

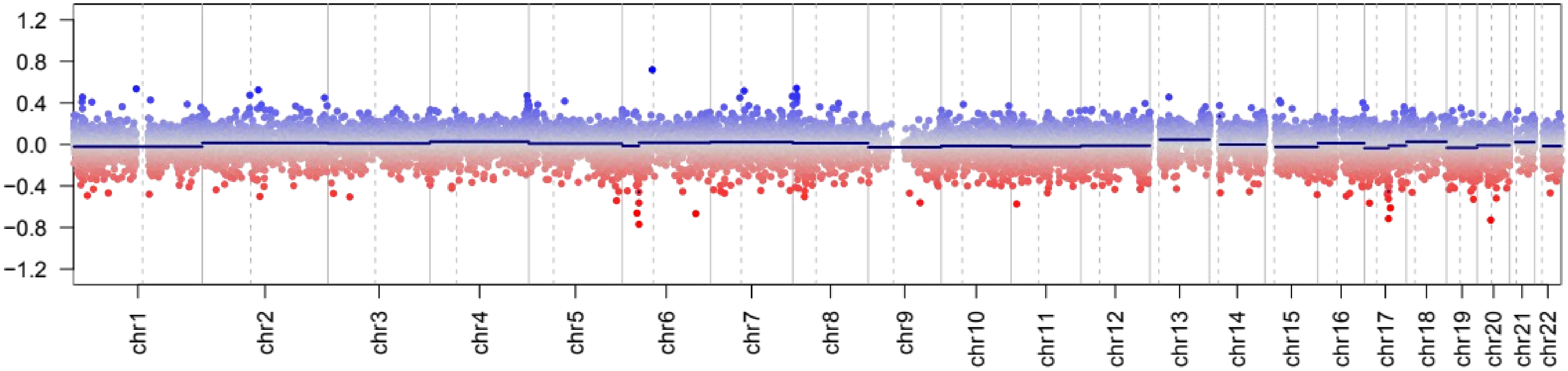

(A) **Ultrasonic aspirator I DX-BLN-054**

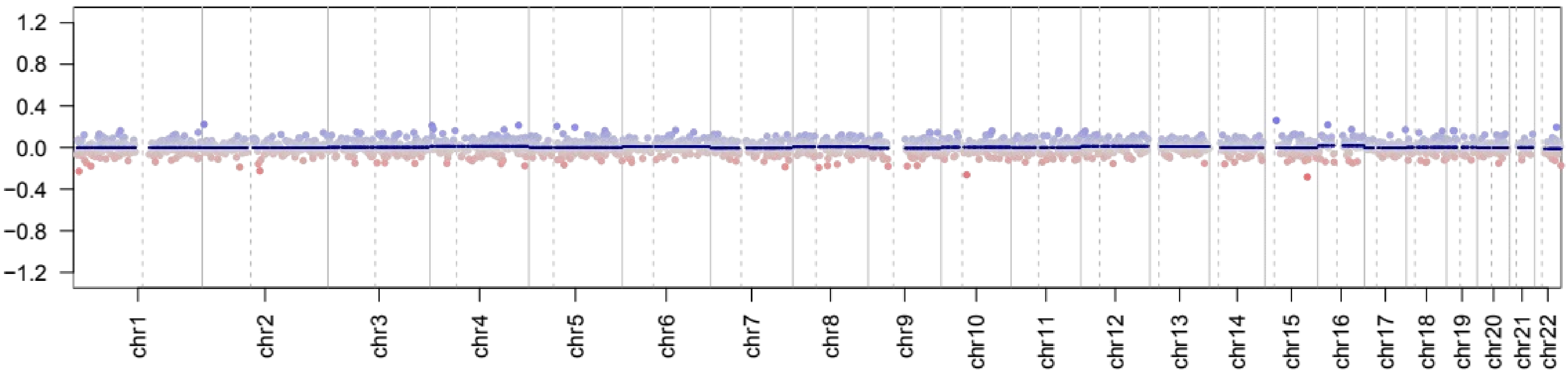

(B) **Microarray I DX-BLN-054**

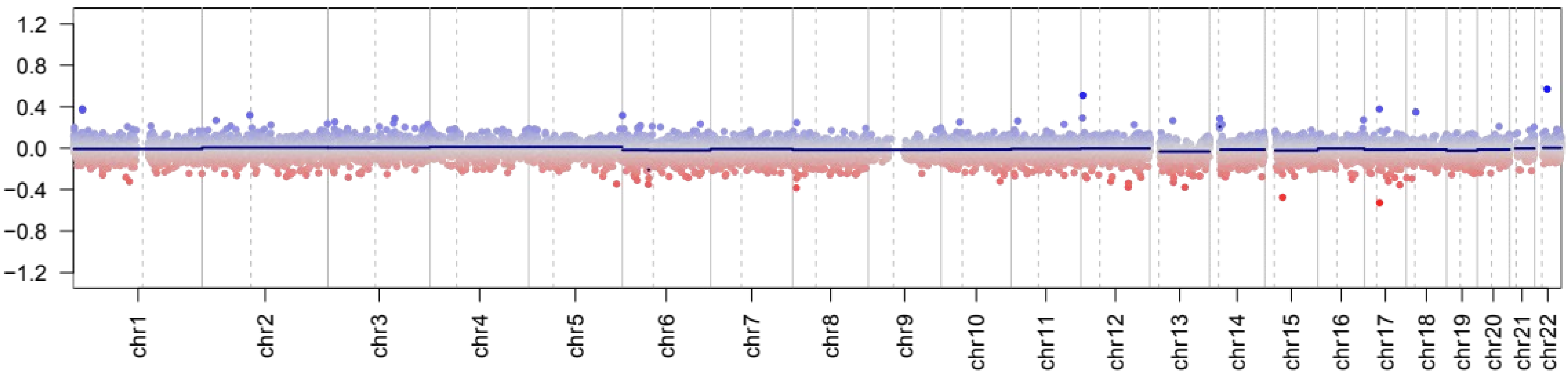

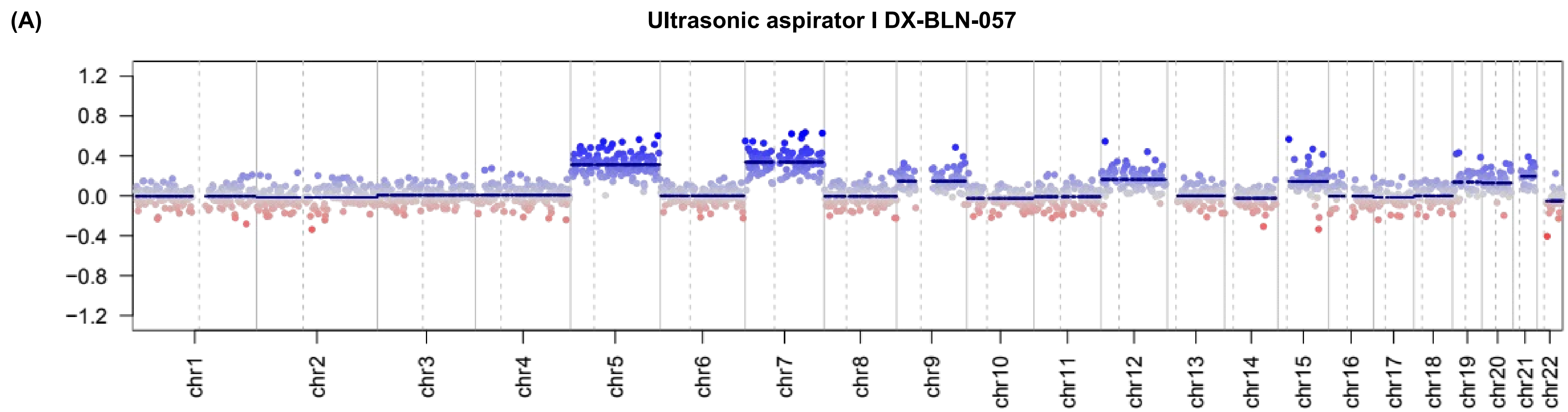
(A)
Ultrasonic aspirator I DX-BLN-057
1.2
0.8
0.4
0.0
−0.4
−0.8
−1.2
chr1 chr2 chr3 chr4 chr5 chr6 chr7 chr8 chr9 chr10 chr11 chr12 chr13 chr14 chr15 chr16 chr17 chr18 chr19 chr20 chr21 chr22

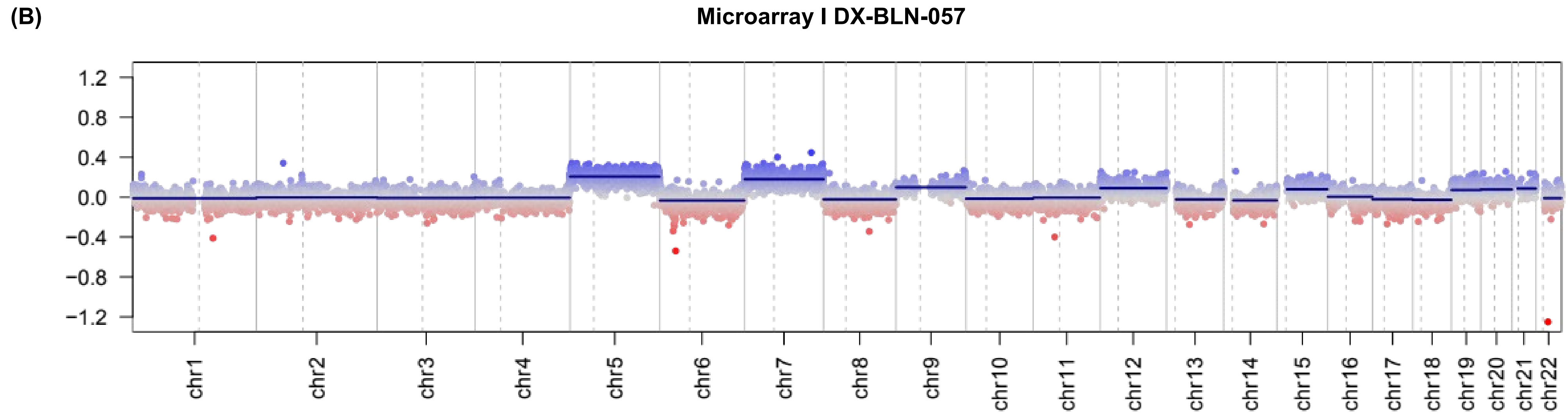
(B)
Microarray I DX-BLN-057
1.2
0.8
0.4
0.0
−0.4
−0.8
−1.2
chr1 chr2 chr3 chr4 chr5 chr6 chr7 chr8 chr9 chr10 chr11 chr12 chr13 chr14 chr15 chr16 chr17 chr18 chr19 chr20 chr21 chr22

(A)

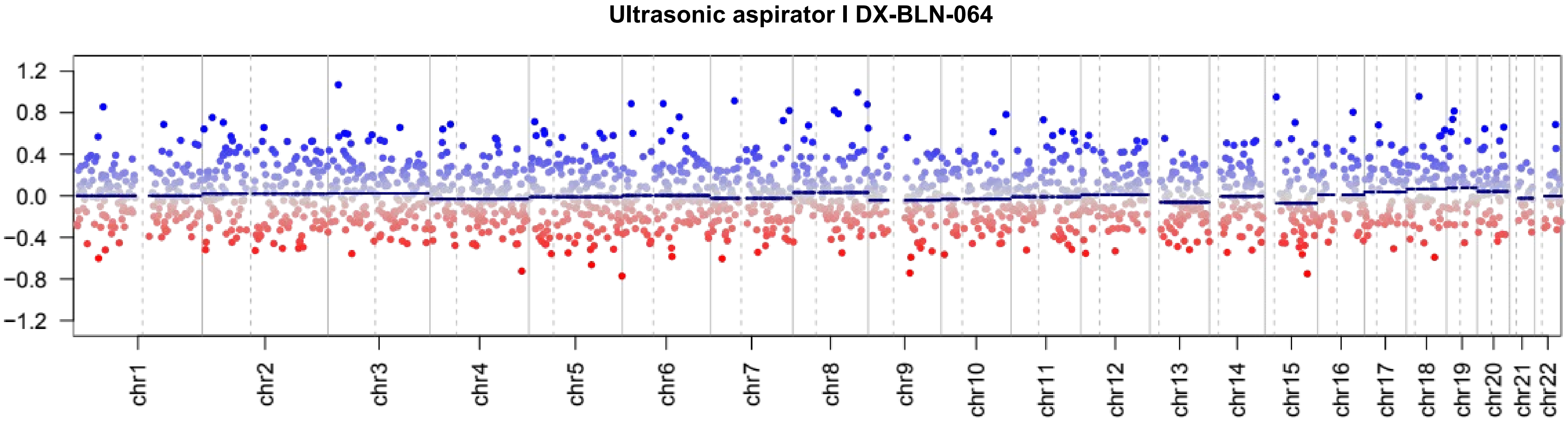
Ultrasonic aspirator I DX-BLN-064
1.2
0.8
0.4
0.0
−0.4
−0.8
−1.2
chr1
chr2
chr3
chr4
chr5
chr6
chr7
chr8
chr9
chr10
chr11
chr12
chr13
chr14
chr15
chr16
chr17
chr18
chr19
chr20
chr21
chr22

(B)

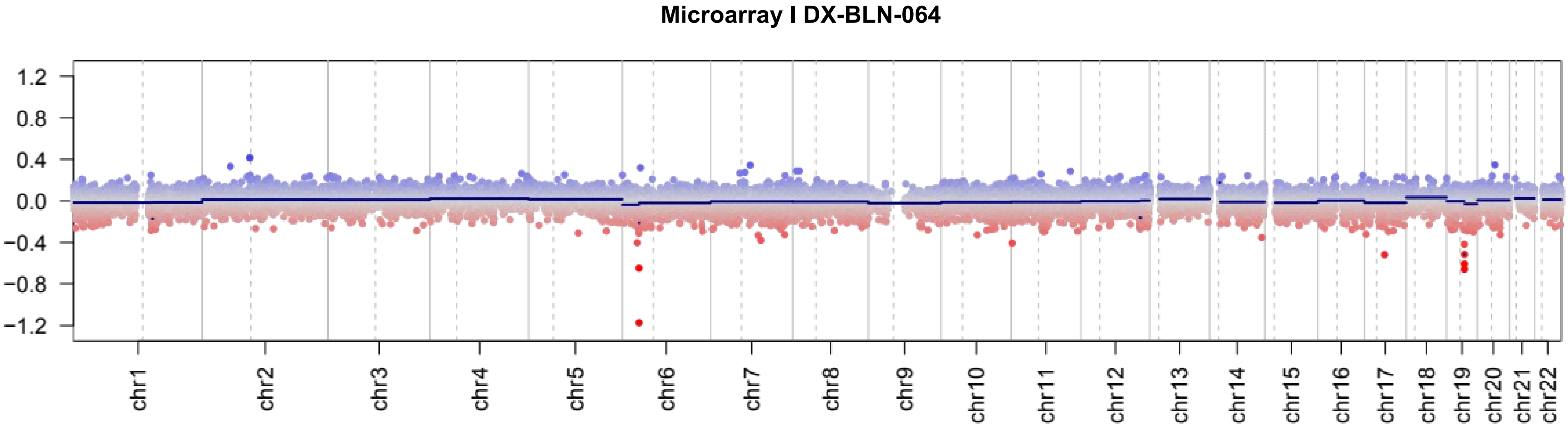
Microarray I DX-BLN-064
1.2
0.8
0.4
0.0
−0.4
−0.8
−1.2
chr1
chr2
chr3
chr4
chr5
chr6
chr7
chr8
chr9
chr10
chr11
chr12
chr13
chr14
chr15
chr16
chr17
chr18
chr19
chr20
chr21
chr22

(A)

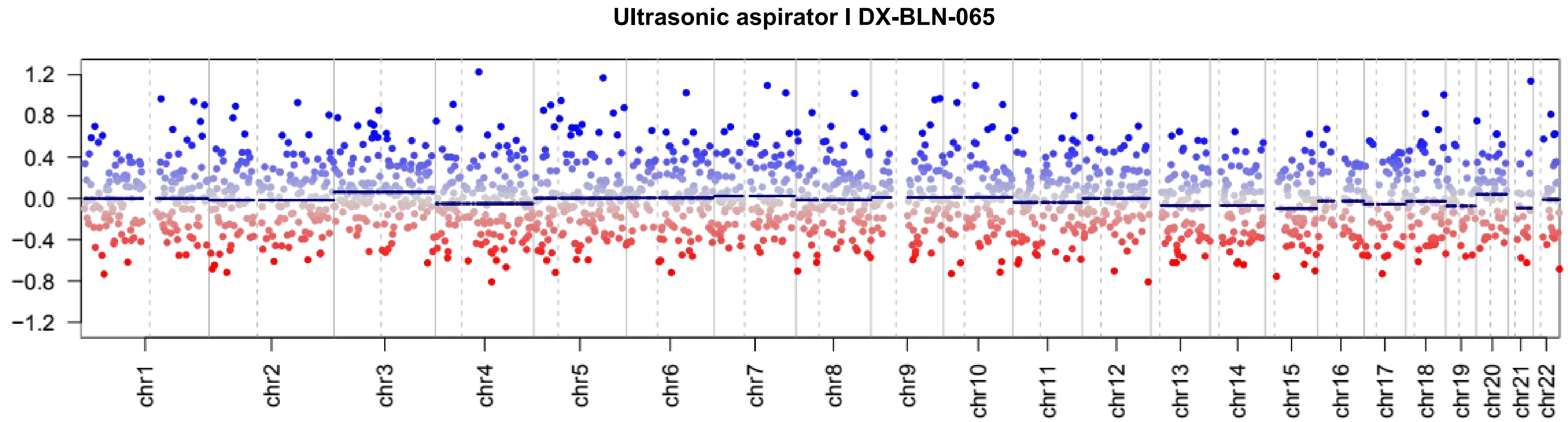
Ultrasonic aspirator I DX-BLN-065
1.2
0.8
0.4
0.0
−0.4
−0.8
−1.2
chr1 chr2 chr3 chr4 chr5 chr6 chr7 chr8 chr9 chr10 chr11 chr12 chr13 chr14 chr15 chr16 chr17 chr18 chr19 chr20 chr21 chr22

(B)

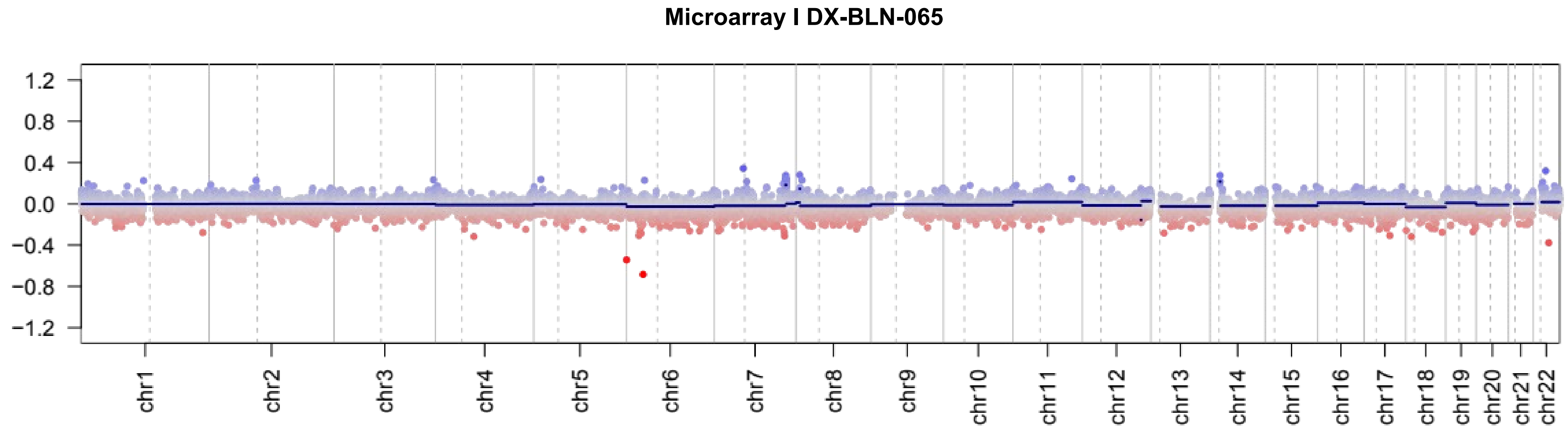
Microarray I DX-BLN-065
1.2
0.8
0.4
0.0
−0.4
−0.8
−1.2
chr1 chr2 chr3 chr4 chr5 chr6 chr7 chr8 chr9 chr10 chr11 chr12 chr13 chr14 chr15 chr16 chr17 chr18 chr19 chr20 chr21 chr22

(A)

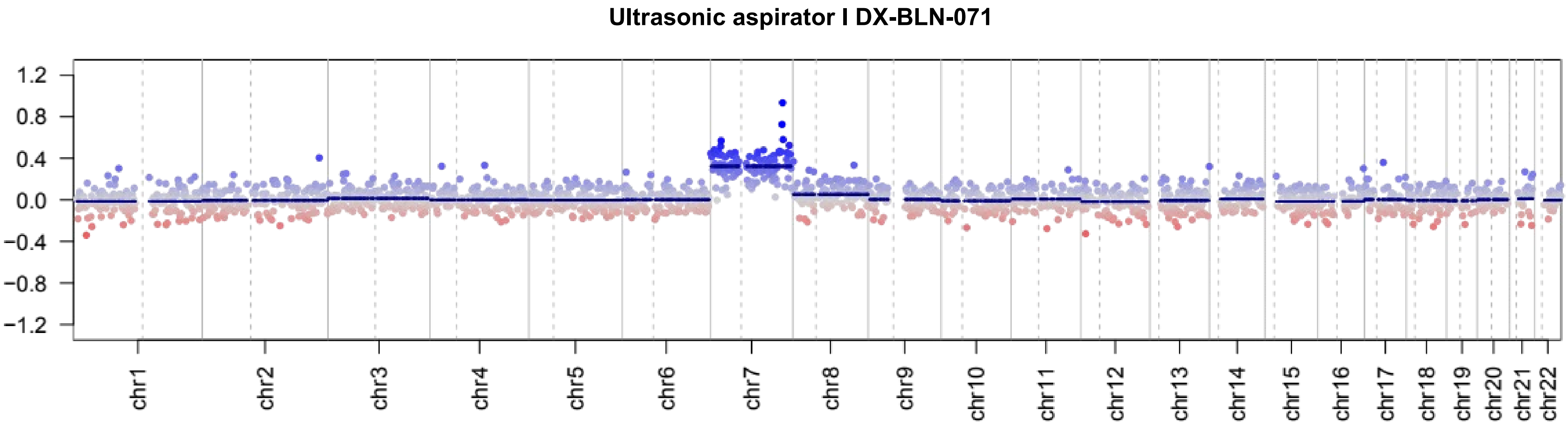

Ultrasonic aspirator I DX-BLN-071
1.2
0.8
0.4
0.0
−0.4
−0.8
−1.2
chr1 chr2 chr3 chr4 chr5 chr6 chr7 chr8 chr9 chr10 chr11 chr12 chr13 chr14 chr15 chr16 chr17 chr18 chr19 chr20 chr21 chr22

(B)

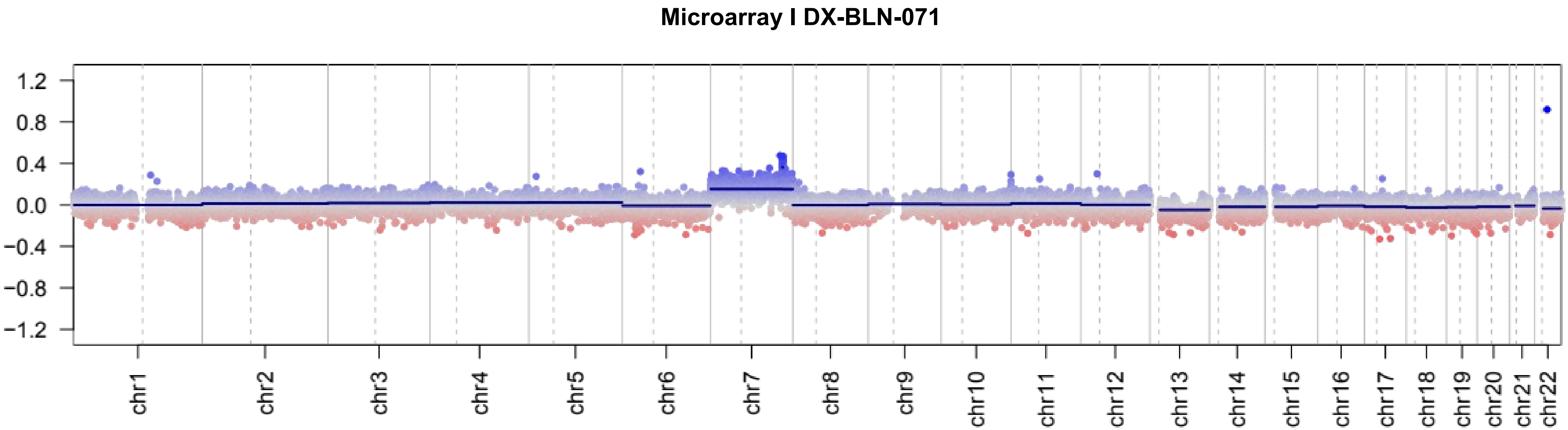

Microarray I DX-BLN-071
1.2
0.8
0.4
0.0
−0.4
−0.8
−1.2
chr1 chr2 chr3 chr4 chr5 chr6 chr7 chr8 chr9 chr10 chr11 chr12 chr13 chr14 chr15 chr16 chr17 chr18 chr19 chr20 chr21 chr22

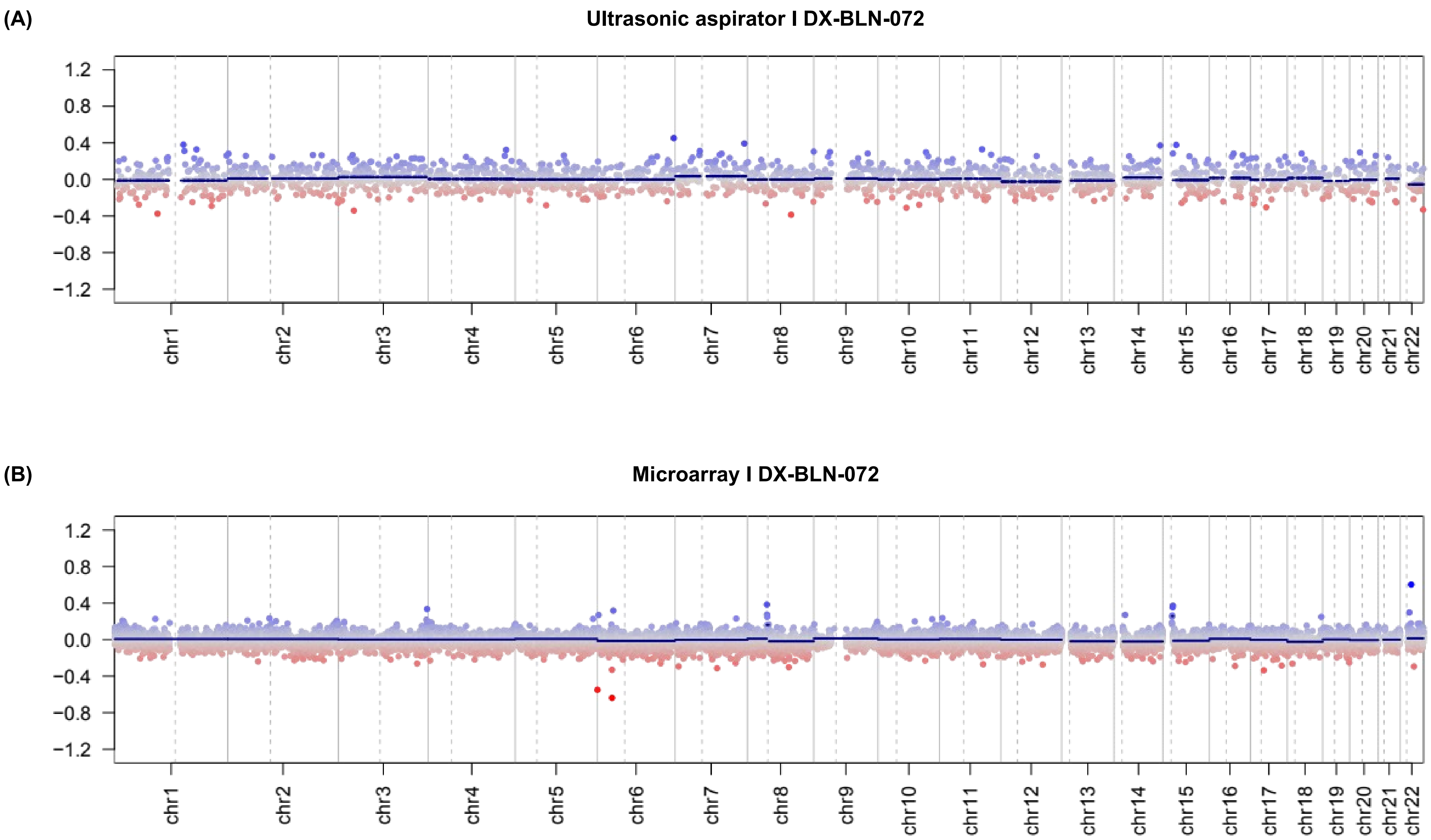

(A)
Ultrasonic aspirator I DX-BLN-072
1.2
0.8
0.4
0.0
−0.4
−0.8
−1.2
chr1 chr2 chr3 chr4 chr5 chr6 chr7 chr8 chr9 chr10 chr11 chr12 chr13 chr14 chr15 chr16 chr17 chr18 chr19 chr20 chr21 chr22
(B)
Microarray I DX-BLN-072
1.2
0.8
0.4
0.0
−0.4
−0.8
−1.2
chr1 chr2 chr3 chr4 chr5 chr6 chr7 chr8 chr9 chr10 chr11 chr12 chr13 chr14 chr15 chr16 chr17 chr18 chr19 chr20 chr21 chr22

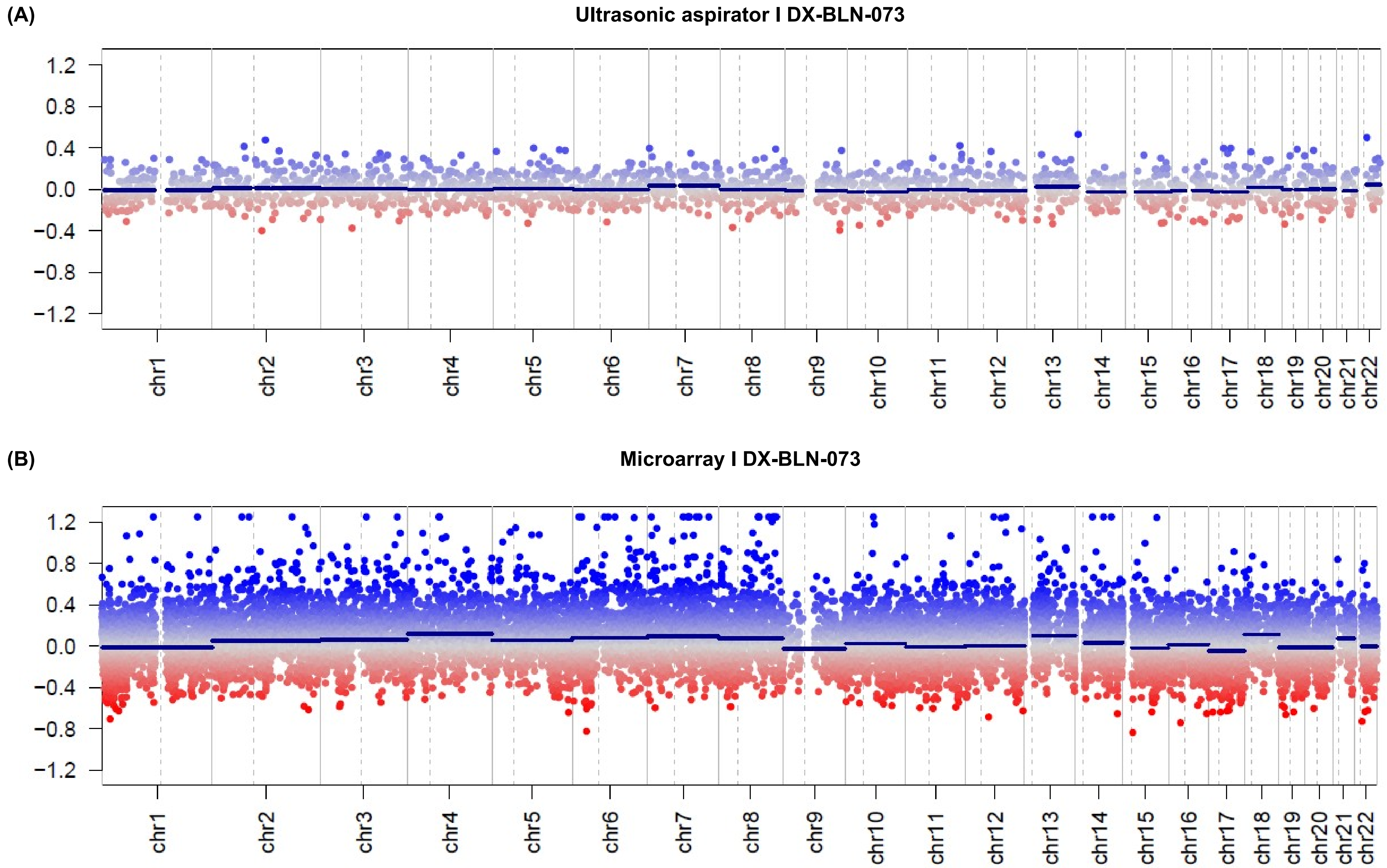
(A)
Ultrasonic aspirator I DX-BLN-073
1.2
0.8
0.4
0.0
−0.4
−0.8
−1.2
chr1
chr2
chr3
chr4
chr5
chr6
chr7
chr8
chr9
chr10
chr11
chr12
chr13
chr14
chr15
chr16
chr17
chr18
chr19
chr20
chr21
chr22
(B)
Microarray I DX-BLN-073
1.2
0.8
0.4
0.0
−0.4
−0.8
−1.2
chr1
chr2
chr3
chr4
chr5
chr6
chr7
chr8
chr9
chr10
chr11
chr12
chr13
chr14
chr15
chr16
chr17
chr18
chr19
chr20
chr21
chr22

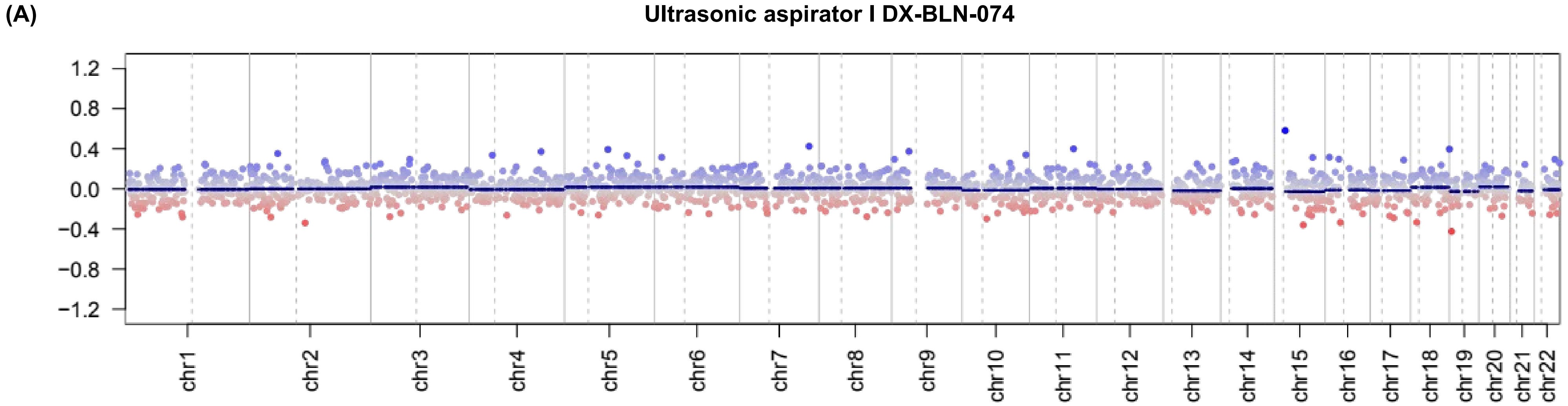
(A)
Ultrasonic aspirator I DX-BLN-074
1.2
0.8
0.4
0.0
−0.4
−0.8
−1.2
chr1 chr2 chr3 chr4 chr5 chr6 chr7 chr8 chr9 chr10 chr11 chr12 chr13 chr14 chr15 chr16 chr17 chr18 chr19 chr20 chr21 chr22

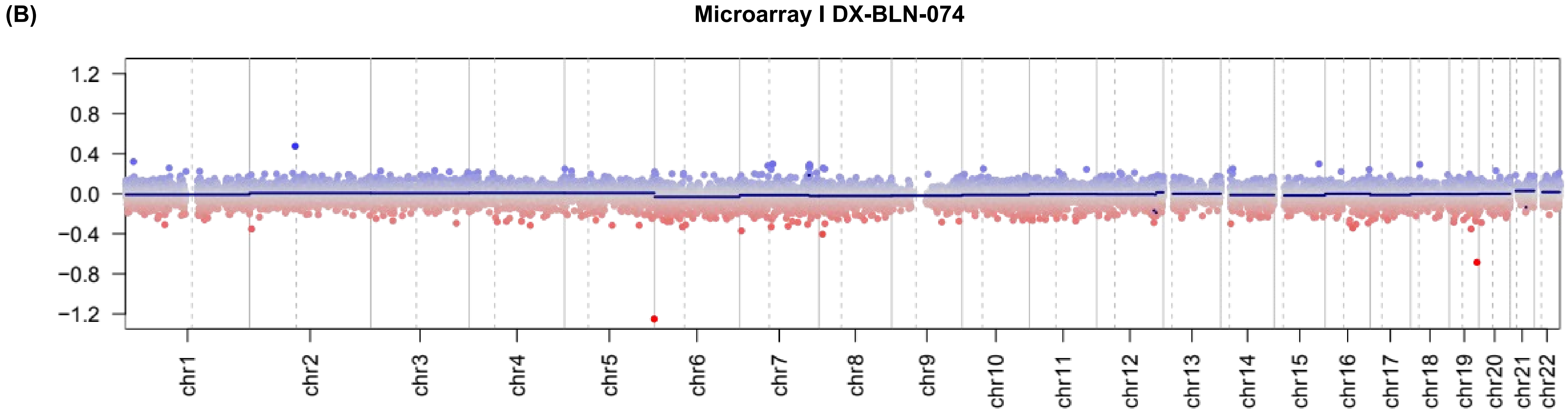
(B)
Microarray I DX-BLN-074
1.2
0.8
0.4
0.0
−0.4
−0.8
−1.2
chr1 chr2 chr3 chr4 chr5 chr6 chr7 chr8 chr9 chr10 chr11 chr12 chr13 chr14 chr15 chr16 chr17 chr18 chr19 chr20 chr21 chr22

(A)

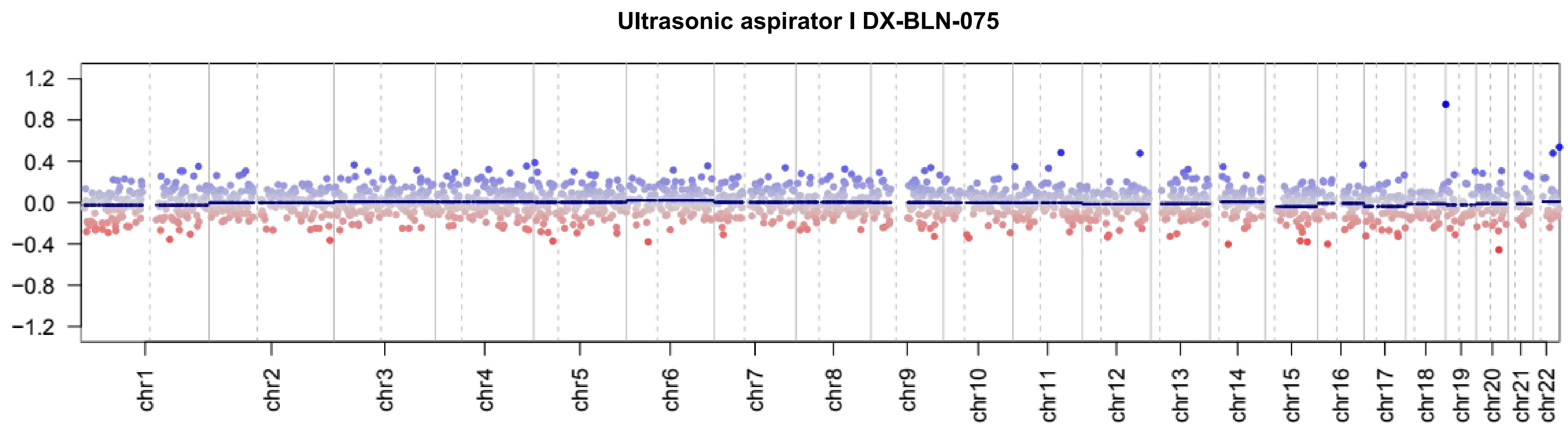
Ultrasonic aspirator I DX-BLN-075
1.2
0.8
0.4
0.0
−0.4
−0.8
−1.2
chr1
chr2
chr3
chr4
chr5
chr6
chr7
chr8
chr9
chr10
chr11
chr12
chr13
chr14
chr15
chr16
chr17
chr18
chr19
chr20
chr21
chr22

(B)

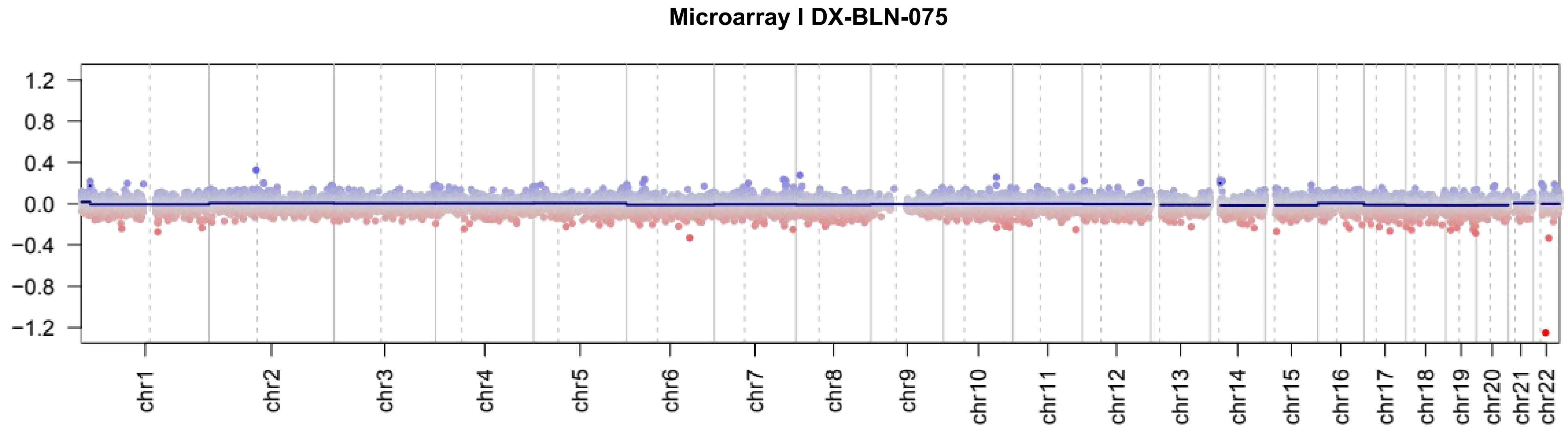
Microarray I DX-BLN-075
1.2
0.8
0.4
0.0
−0.4
−0.8
−1.2
chr1
chr2
chr3
chr4
chr5
chr6
chr7
chr8
chr9
chr10
chr11
chr12
chr13
chr14
chr15
chr16
chr17
chr18
chr19
chr20
chr21
chr22
